# Acute Effects of Dairy or Soy Milk on Sex Hormones Following Resistance Exercise in Males: A Randomized, Crossover Pilot Trial

**DOI:** 10.7759/cureus.59972

**Published:** 2024-05-09

**Authors:** Joel C Craddock, Amelia Wakefield, Gregory E Peoples, David M Goldman, Theresa A Larkin

**Affiliations:** 1 School of Medical, Indigenous and Health Sciences, Faculty of Science, Medicine and Health, University of Wollongong, Wollongong, AUS; 2 Department of Public Health, Faculty of Medicine, University of Helsinki, Helsinki, FIN; 3 Research and Development, Metabite, Inc., New York, USA; 4 Graduate School of Medicine, Faculty of Science, Medicine and Health, University of Wollongong, Wollongong, AUS

**Keywords:** lactose intolerant, plant-based milk, progesterone, estrogen, testosterone, hormones, strength training, soy milk, dairy milk

## Abstract

Introduction: Resistance exercise training (RET) can increase muscle mass and strength, and this adaptation is optimized when dietary protein is consumed to enhance muscle protein synthesis. Dairy milk has been endorsed for this purpose; however, allergy and lactose intolerance affect two-thirds of the global population making dairy milk unsuitable for many. Plant-based alternatives such as soy milk have gained popularity and exhibit comparable protein content. However, concerns regarding soy phytoestrogens potentially influencing circulating sex hormones and diminishing the anabolic response to RET have been raised. This study therefore aimed to assess the acute effects of dairy and soy milk consumption on circulating sex hormones (total, free testosterone, free testosterone percentage, total estrogen, progesterone, and sex hormone binding globulin) after RET.

Materials and methods: Six male participants were recruited for a double-blinded, randomized crossover study with either dairy or soy milk provided post RET. Venous samples were collected before and after milk consumption across seven timepoints (0-120 minutes) where circulating sex hormones were analyzed. Two-way ANOVA analyses were applied for repeated measures for each hormone. The area under the curve (AUC) was also calculated between dairy and soy milk. Significance was set at p<0.05.

Results: No significant differences were observed in acute circulating serum for free (p=0.95), % free (p=0.56), and total testosterone (p=0.88), progesterone (p=0.67), or estrogen (p=0.21) between milk conditions. Likewise, no significant differences in AUC were observed between any hormones.

Conclusion: These findings suggest that consumption of dairy milk and soy milk have comparable acute effects on circulating sex hormones following RET. Further investigations with expanded sample sizes are needed to strengthen and broaden these initial findings.

## Introduction

Resistance exercise training (RET) is widely adopted by recreational exercisers and competitive athletes to improve skeletal muscle strength and mass, as well as overall exercise performance. During the acute period following RET (within one hour), protein consumption is recommended to facilitate muscle adaptations and recovery [1]. Dairy milk has been endorsed in this context due to its considerably high protein concentrations, most of which are casein and whey [2]. However, dairy milk allergy is the most prevalent food allergy, affecting up to 17% of the global population [3] and lactose intolerance affects two-thirds of the global population [4], making dairy unsuitable for many individuals. Dairy milk consumption has been steadily declining in Europe and the United States [5]. Plant-based milks provide an alternative to dairy milk and have been recently gaining popularity, and one of the most commonly used substitutes is soy milk, especially in exercising individuals [6]. Regular exercise increases protein requirements, and dairy and soy milk exhibit comparable total protein contents [7]. Studies have also shown comparable effects on muscle strength adaption for whey and soy protein [8,9]. However, perceived risks surrounding the effect of soy on sex hormones and their links to ‘feminization’ in men may discourage consumption [10].

The sex hormones testosterone, progesterone, and estrogen can influence anabolic pathways, adaptations to RET, and body composition. Testosterone activates satellite cells and promotes myonuclear accretion, facilitating muscle fiber hypertrophy [11]. Exercise-induced testosterone elevations have been shown to translate into increased muscle hypertrophy and strength development [12], while suppression of endogenous testosterone production diminishes the anabolic response to RET [13]. Elevated progesterone levels have been shown to depress serum testosterone levels and reduce thigh muscle cross-sectional area, exerting an anti-anabolic effect on muscle even when combined with exogenous testosterone in the context of RET [14]. Higher progesterone has also been shown to promote weight gain that is mostly or entirely fat in males and females [15]. Estrogen has been shown to improve muscle mass, strength, and collagen content, yet, in tendons and ligaments, estrogen can decrease stiffness, affecting performance and increasing injury rates [16]. While these hormones clearly influence anabolic pathways and body composition, research examining estrogen and progesterone levels post RET has predominantly been conducted in female cohorts [17,18]. Conversely, research analyzing anabolism following acute RET in men has typically focused on testosterone, growth hormone, insulin-like growth factor, and cortisol [19], thereby highlighting a gap in the body of evidence pertaining to estrogen and progesterone in men following RET.

Concerns about the effects of soy on sex hormones and the ensuing anabolic response to RET largely surround the isoflavones. These are a class of phytoestrogens found naturally in many plant foods and particularly concentrated in soy [20]. Isoflavones act as selective estrogen receptor modulators, exerting estrogen-like effects in some tissues and anti-estrogenic effects in others [21]. However, the effects of isoflavones are not equivalent to those of endogenous estrogens due to multiple biochemical differences [22]. For instance, while isoflavones share a similar chemical structure allowing them to bind to estrogen receptors, their effects vary and, in some instances, have been estimated to be some thousandfold weaker compared to 17-beta estradiol (E2), the primary female sex hormone [23]. In addition, their binding affinity to the two estrogen receptors differs when compared to estrogen resulting in divergent physiological outcomes such as transcriptional expression [24]. However, many athletes who engage in resistance training remain concerned about the potential effects of soy on sex hormones [21]. "Feminizing effects" such as gynecomastia are rare but have been documented when consuming very large quantities (2.8 liters per day) of soy milk [25].

While soy and isoflavones have come under scrutiny with respect to phytoestrogens, it is important to note that no estrogens or progesterone have been detected in soy [21,26]. The testosterone contents of soy are also unremarkable and comparable to levels detected in other plant foods [21]. In contrast, dairy milk and dairy products supply 60-80% of ingested female sex steroids [27]. Modern dairy milk production has undergone significant changes over the past decades, with the aim of maximizing efficiency and commercial output. Natural reproductive cycles in cows involve pregnancy followed by lactation to suppress ovulation and conception. To optimize commercial milk output, cows are artificially inseminated and kept pregnant for most of their lactation period, allowing for nearly continuous milk harvesting [28]. While this approach benefits farmers by increasing milk production, it leads to alterations in the composition of dairy milk available to consumers. As cows spend more time pregnant during lactation, sex-steroid-related pregnancy hormones accumulate in the milk. Concentrations of free and enzymatically deconjugated estrone (E1) rise significantly throughout the course of pregnancy, increasing from 7.9 ng/L in non-pregnant cows to 1266 ng/L during the third trimester of pregnancy [29]. Concentrations of 17 alpha-E2 and 17 beta-E2 also exhibit a notable increase during pregnancy, rising from 33 to 322 ng/L and from 18.6 to 51.2 ng/L, respectively [29]. Consequently, human consumers are exposed to these hormones when consuming dairy milk [28].

As dairy milk contains sex hormones and soy milk contains molecules that are structurally similar to sex hormones, these beverages have the potential to influence circulating sex hormones and subsequently muscle adaptations to exercise. The purpose of this study was to examine and compare the acute effects of dairy milk and soy milk consumption on serum sex hormone concentrations following an acute bout of RET. It was hypothesized that dairy milk consumption following resistance exercise will result in higher circulating estrogen and progesterone levels and lower circulating free and total testosterone levels compared to soy milk.

## Materials and methods

The study utilized a double-blinded, randomized, within-subject repeated measures, crossover design at the University of Wollongong, Wollongong, Australia. Consent was obtained from all participants before the study commencement. It was approved by the University of Wollongong’s Human Research Ethics Research Committee (2022/211). The study was registered with the Australian New Zealand Clinical Trials Registry (registration number: ACTRN 12622001363774). It was conducted in accordance with the Code of Ethics of the World Medical Association (Declaration of Helsinki).

Participants attended the laboratory at the University of Wollongong on three occasions The initial visit was a familiarization and maximal strength assessment. The maximal strength assessment occurred for the following exercises: barbell bench press, barbell squats, seated barbell shoulder press, standing barbell bicep curls, and pronated pull-ups and was used to determine one-repetition maximum (1-RM) of each participant. The crossover occurred during the second and third visits where three sets of each exercise were performed at 70% of participants 1-RM until voluntary failure. At least 48 hours separated visits two and three, which were both completed within a seven-day time frame. Blood was collected immediately following exercise at visits two and three. Participants were then randomly allocated to consume dairy milk or soy milk at visit two and the alternate milk at visit three. Blood collection occurred at six timepoints after milk consumption.

Participants

Male participants aged between 18 and 30 years were recruited for this study. Invitations to participate were posted to local relevant social media groups. The inclusion criteria for participants consisted of engagement in regular resistance exercise for at least two years with a frequency of at least three times per week, a body mass index (BMI) between 18.5 and 30 kg/m^2^, and a level of English proficiency suitable to provide informed consent. The exclusion criteria for participants included the presence of any lactose or soy intolerance or allergy, any cardiac, hepatic, pulmonary, renal, neurological, hematological, psychiatric, or gastrointestinal illness, the use of ergogenic aids (i.e. anabolic steroids), and participants who were taking any medications. Screening was conducted to ensure participant suitability prior to study commencement. Participant exercise readiness was obtained using a pre-existing exercise readiness questionnaire [30]. Participants were required to read a participant information sheet and were given the opportunity to discuss any concerns prior to their involvement in the study. Informed written consent was subsequently obtained. 

Procedures

Visit One: 1-RM Assessment

At the initial visit, participant height (m) and body mass (kg) were recorded using a stadiometer (Seca 217 Stable Stadiometer; seca GmbH & Co. KG., Hamburg, Germany) and calibrated electronic scales (Seca 875 Flat Scale; seca GmbH & Co. KG.) under laboratory conditions. Each participant performed a warm-up of five minutes walking on a treadmill at 6 km/hour. Following this, participants performed a maximal strength assessment to determine their 1-RM for the following exercises: barbell bench press, seated barbell shoulder press, barbell squats, standing barbell bicep curls, and pull-ups. Initially, participants were asked for their anticipated 1-RM weight for each exercise. They proceeded to warm up by completing 5-10 repetitions at 50% of their anticipated 1-RM for each exercise. Following a rest period of two minutes, participants completed two to three repetitions at 70-80% of their anticipated 1-RM load. Weight was then increased gradually by approximately 5-10% until failure, with the 1-RM determined as the heaviest successful attempt out of four attempts at each exercise. Two-minute rest periods were provided between attempts. The determined 1-RM was then used to calculate the 70% workload for each participant for visits two and three.

Visits Two and Three: Acute RET and Milk Consumption

In the 48 hours prior to visits two and three, intake of dairy, soy, and their derivative products as well as alcohol and more than one cup of coffee was prohibited. Participants were asked to photograph all food and beverage intake to monitor adherence using their smartphones. Exercise in the 48 hours prior to visits two and three was also not permitted. In the 12 hours prior to visits two and three, participants were instructed to fast. Water consumption was allowed throughout the study including during fasting periods and during exercise. Outside of these time periods, participants could engage in their regular diet and exercise regimes. An accredited practicing dietitian was available to advise and answer questions related to diet throughout the entire study.

Training Sessions

All participants completed the RET sessions at the same time of day (between 0700 and 0900 hours) to control for diurnal hormonal fluctuations. On visits two and three to the laboratory, identical resistance exercise protocols were performed, which were adapted from Simao, et al. [31], the intervention that elicited the largest acute rise in free testosterone following physical exercise in a recent meta-analysis [32]. Participants warmed up by walking on a treadmill for five minutes at 6 km/hour. They proceeded to perform the same exercises completed in the 1-RM testing (barbell bench press, seated barbell shoulder press, barbell squats, standing barbell bicep curls, and pull-ups) at 70% of their 1-RM until voluntary failure. Two minutes of passive rest were provided between sets and different exercises. Pausing between the eccentric and concentric phases of each repetition was not permitted.

Nutritional Conditions: Dairy Milk and Soy Milk Consumption

Utilizing a double-blinded randomized (block) crossover design, participants underwent each treatment condition, consuming dairy milk and soy milk following RET. The dairy milk was organic, grass-fed, locally procured whole milk. The soy milk was also organic, made from whole soybeans and water. 600 mL/m^2^ (approximately 1100 mL total) of milk was provided, a quantity administered in previous research yielding 33-40 g protein [33]. This translated to 0.48-0.56 g/kg protein (for men weighing 59.0-83.0 kg), an amount that aligns with the recommendation to consume 0.4-0.55 g/kg protein per meal to maximize anabolism [34]. Milk was provided at +45 minutes post-RET and was consumed within 10 min to ensure intake within the “post-workout anabolic window of opportunity,” which is generally considered to be within an hour after completing RET [1].

Blood Collection and Hormone Analysis 

At both RET sessions (visits two and three), 4 mL of blood was collected at each of seven time points: directly following exercise completion, and at 30, 45, 60, 80, 100, and 120 minutes following milk consumption. These time points were informed by previous research investigating dairy and soy milk intake on sex hormones and the included response [33]. Blood was collected by a trained phlebotomist and samples were held for 20-30 minutes before being centrifuged at 3500 rpm at 4 degrees Celsius for 10 minutes. Serum was then extracted from the separated blood sample and pipetted into Eppendorf tubes which were then stored at -80 degrees Celsius until analysis. Cardinal laboratory analyzed the serum for total, free testosterone, free testosterone percentage, total estrogen, progesterone, and sex hormone binding globulin (SHBG).

Statistical analyses

Analysis of the collected data was conducted using GraphPad Prism version 8.0 (Dotmatics, Boston, Massachusetts, United States) software. The Shapiro-Wilks test was used to test for normality. Paired t-tests were used to compare differences between the mean number of repetitions lifted and total mass moved between visits two and three. Two-way ANOVA analyses were applied for repeated measures (total testosterone, free testosterone, free testosterone percentage, total estrogen, progesterone, and SHBG) with milk condition and time as the two variables with Bonferroni post hoc analysis performed. The area under the curve (AUC) was determined between dairy and soy milk for each hormone. The data collected were expressed as mean and standard deviation (SD). Alpha was set at p<0.05.

## Results

**Figure 1:**
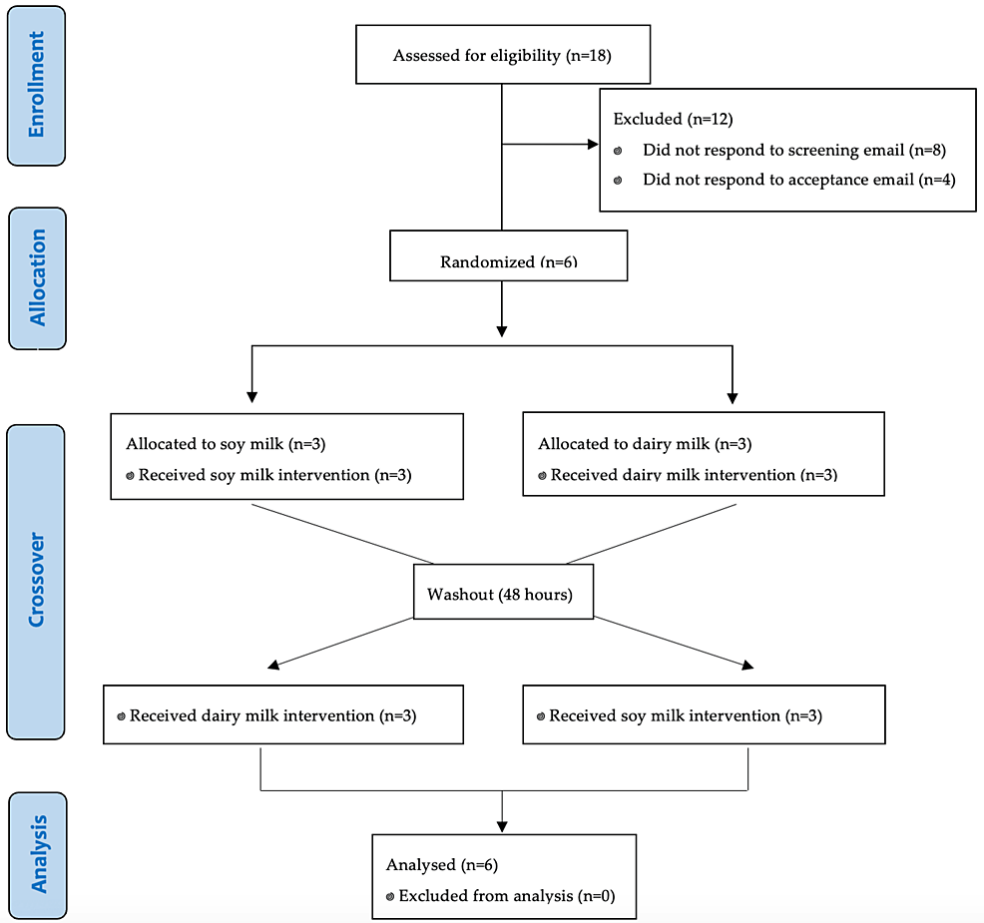
CONSORT flow diagram depicting the flow of participants through the randomized crossover trial. CONSORT: Consolidated Standards of Reporting Trials

There were 18 individuals who expressed initial interest in participating in the study between November 2022 and April 2023. Of the 18 interested individuals, eight did not respond to the screening email. Among the remaining 10 eligible participants, four individuals did not respond to the acceptance email, resulting in a final enrollment of six participants who successfully completed the study (Figure 1). Participants were aged between 18 and 28 years, with a height range of 1.77-1.91 m and a body mass range of 68.1 kg to 107.4 kg (Table 1). BMI ranged from 19.9 to 29.7 kg/m^2^. Participants reported adherence to refraining from exercise in the 48 hours prior to visits two and three. Similarly, all participants had omitted soy and dairy in the 48 hours prior to visits two and three, which was confirmed by photographed meals reviewed by the accredited practicing dietitian. 

**Table 1:** Participant data

Participants	Age (years)	Height (m)	Weight (kg)	BMI (kg/m^2^)	Training experience (years)
1	28	1.91	107.4	29.4	10
2	21	1.87	103.7	29.7	2
3	24	1.77	73	23.3	3
4	21	1.79	72	22.5	3
5	18	1.87	86.7	24.8	3
6	18	1.85	68.1	19.9	3
Mean ± standard deviation	21.67±3.83	1.84±0.05	85.15±17.05	24.93±3.91	4±2.9

RET

There were no significant differences between total mass moved for participants between visits two and three for shoulder press (p=0.18), bicep curl (p=0.65), bench press (p=0.37), pull-ups (p=0.42), squats (p=0.21), or all exercised combined (p=0.94).

Sex hormones

**Figure 2:**
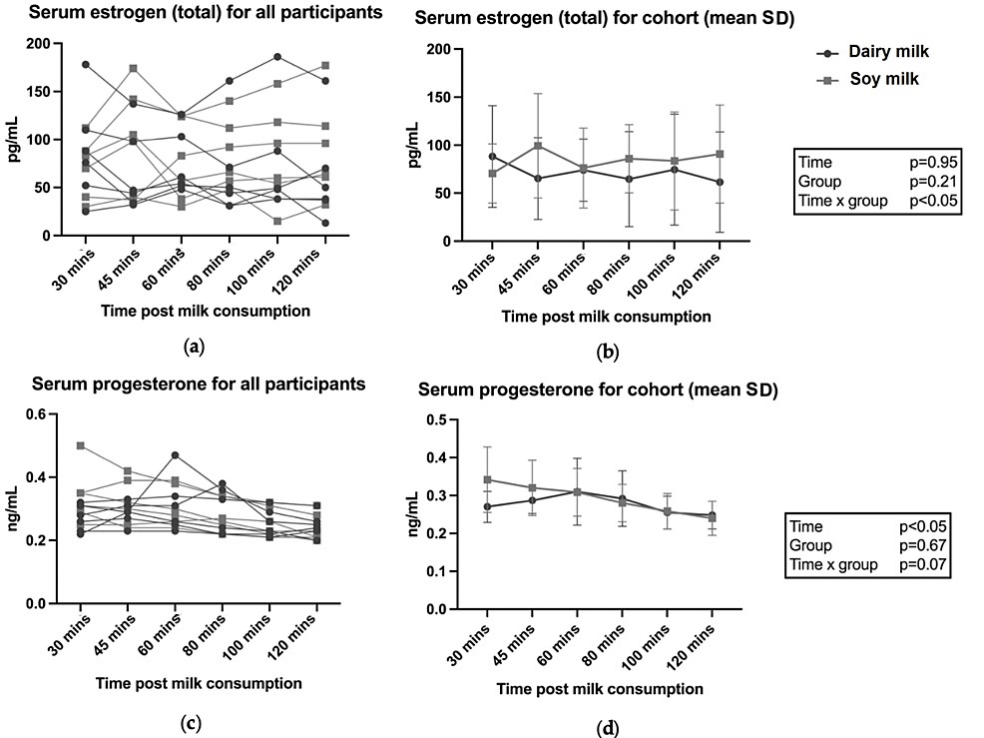
Serum estrogen (total) and progesterone following dairy and soy milk consumption for individuals and the cohort (mean and SD). P-values reported from two-way ANOVA analysis

**Figure 3:**
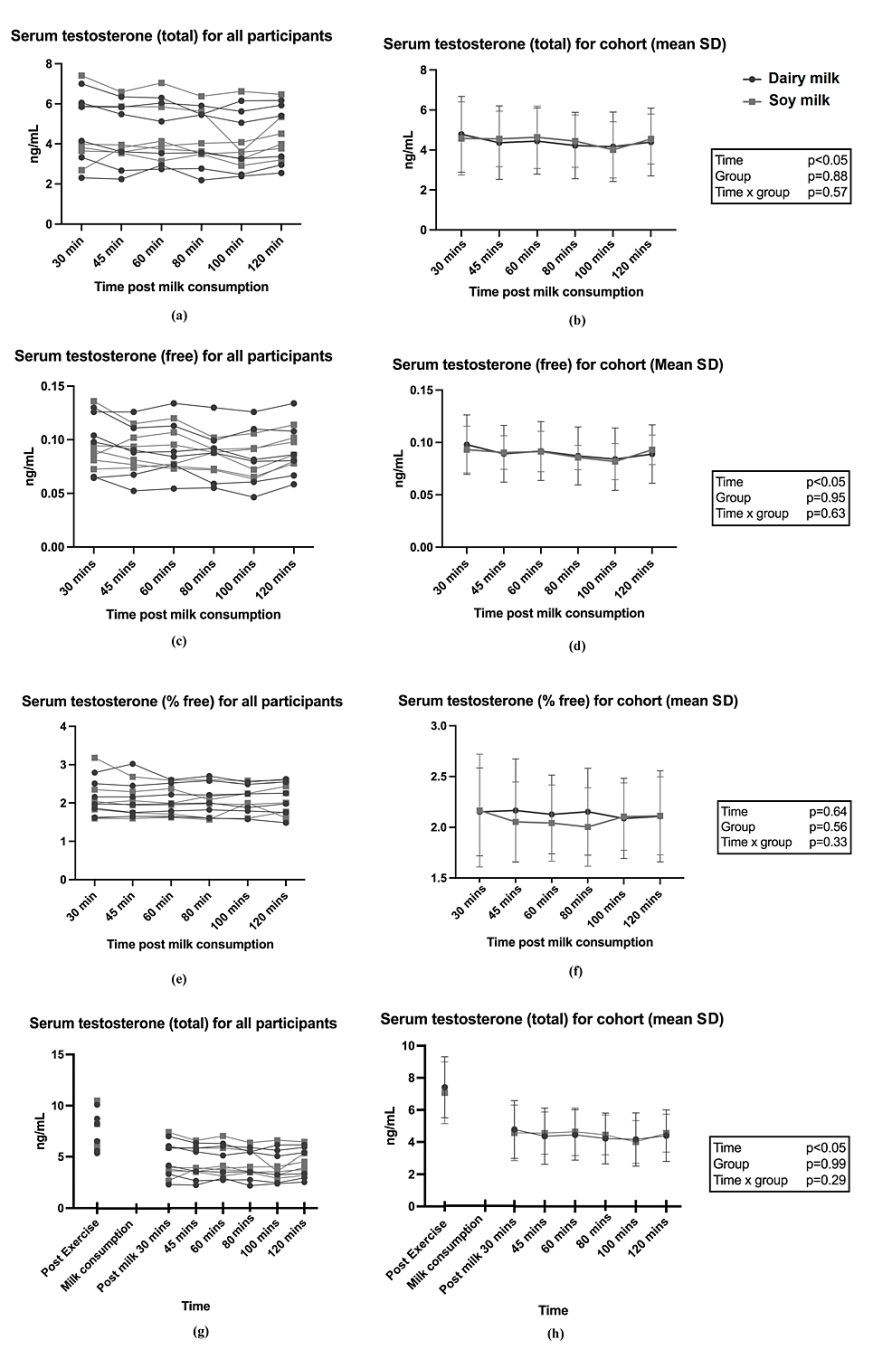
(a-f) Testosterone (total, free, percentage free) following dairy and soy milk consumption for individuals and groups (mean and SD); (g, h) Testosterone directly after RET and following milk intake. RET: resistance exercise training P-values reported from two-way ANOVA analysis

**Figure 4:**
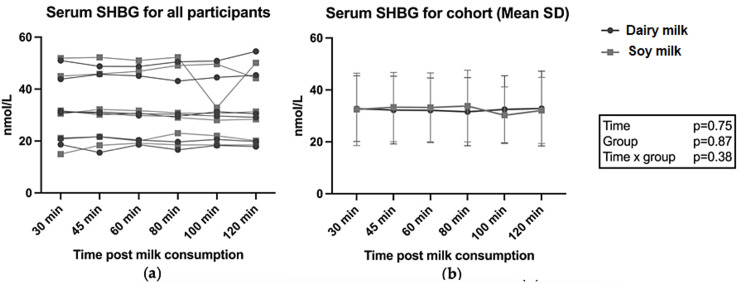
Serum SHBG following dairy and soy milk consumption for individuals and groups (mean and SD). SHBG: sex hormone binding globulin P values reported from two-way ANOVA analysis

AUC for all hormones between conditions were not statistically different between the two milk groups (P>0.05; data not shown). Figure 2 shows the results for individual and group data for estrogen (total) and progesterone across milk conditions. There was a significant time x milk effect for total estrogen following soy milk consumption (p<0.05) (Figure 2b); however, the post-hoc analysis did not identify time-relevant differences between the groups. Testosterone results are shown in Figure 3. Total testosterone was elevated immediately following RET, with a significant time effect observed after both dairy milk and soy milk (p<0.05) (Figure 3h), but there were no differences between the two milk conditions. No differences were observed in SHBG following dairy and soy milk consumption (Figure 4). Descriptive statistics between milk consumption at each time point for total estrogen, free testosterone, total testosterone, free testosterone, progesterone, and SHBG can be seen in the Appendices.

## Discussion

To our knowledge, this is the first study that has compared the effects of dairy and soy milk consumption as a recovery beverage following RET on acute circulating sex hormones. Free and total testosterone levels were consistent with other studies reporting elevated serum concentrations immediately following exercise which provides context for investigating the presence of sex hormones in circulation [32]. There was an equivalence between total repetitions and total mass moved during RET for both conditions, providing a suitable model to compare levels of circulating sex hormones. The results of this pilot study suggest that there is no difference in circulating serum sex hormones following consumption of soy versus dairy milk. Given the pivotal role of testosterone in muscle protein synthesis (MPS), the finding that soy milk consumption after RET did not result in lower circulating testosterone levels compared with dairy milk is an important finding. While there was a group x time interaction effect for total estrogen, the post-hoc analysis did not identify time-relevant differences between the two milk conditions, and no differences in AUC for any hormones were observed. Overall, the findings suggest equivalence in acute circulating serum sex hormone concentrations after soy and dairy milk consumption.

Contrary to our hypothesis and other research showing dairy milk consumption can elevate circulating estrogen levels, this was not observed in the present study. It was hypothesized that dairy milk consumption following resistance exercise would produce a greater increase in circulating estrogen compared to soy milk due to the high concentrations of sex hormones typically found in commercially available dairy milk [27]. This hypothesis was also formulated with the understanding that hormones found in commercial dairy milk translate into elevated concentrations of circulating sex hormones in humans. For example, in a study involving a cohort of seven adult men and seven children, the consumption of 600 mL/m^2^ of whole milk resulted in significant increases in urinary levels of E1, E2, E3, and pregnanediol, measured by spot urine samples, and serum estrone and progesterone were also significantly elevated [33]. Similarly, Michels et al. examined urinary excretion of sex hormones following either skim (1.5% fat) or whole milk (3.5% fat) consumption in a crossover intervention of 109 postmenopausal women [27]. They observed that the consumption of dairy milk leads to a significant rise in E1 while ingestion of semi-skimmed milk appeared to significantly elevate E2 and E3. Furthermore, Carruba et al. conducted an interventional trial wherein 100 healthy postmenopausal women were assigned to follow either a Mediterranean diet or a Western diet, the latter being characterized by a high intake of milk and dairy products, meat, and animal fat [35]. The findings revealed that participants adhering to the Mediterranean diet experienced more than 40% reduction in urinary estrogen levels compared to those following the Western diet. However, while these studies all reported elevations in estrogens following dairy milk, they either did not have a control group or their comparator group consumed no milk, which was disparate to the present pilot study.

It was also hypothesized that testosterone would be lower following dairy milk versus soy milk consumption due to the high concentrations of progesterone typically found in commercially available dairy milk [36], which can depress serum testosterone levels [14]. A study by Maruyama et al. including seven men and seven children observed a significant reduction in serum testosterone concentration following consumption of 600ml/m^2^ dairy milk [33]. However, this was not observed in the present study whereby both free and total testosterone and progesterone were equivalent between milk conditions. This finding is noteworthy because testosterone is an androgenic anabolic hormone, exerting a wide range of biological effects including the stimulation of muscle growth [12]. Considering that free and total testosterone concentrations and free testosterone percentage were equivalent between groups, it is likely that the serum sex hormone profiles following consumption of both milks would produce a similar anabolic effect in response to RET.

Protein considerations between milk types

With regards to protein intake and its effect on the anabolic response, the "leucine threshold" theory suggests that the magnitude of the rise in blood leucine levels after a meal plays a crucial role in controlling the extent of the post-meal MPS response to the protein source consumed [37]. When a sufficient amount of total protein is consumed and leucine requirements are met, the type of protein may be inconsequential for anabolic and ergogenic outcomes [38]. Evidence in support of this theory can be observed in several studies comparing varying protein types (soy, whey, rice, pea), whereby providing a protein dose that sufficiently meets the "leucine threshold" results in equivalent increases in muscle mass and strength regardless of protein origin [39-42]. While there are limited studies that have directly compared the effects of dairy and soy milk on strength and MPS, two studies have demonstrated modest enhancements in hypertrophy in individuals consuming dairy milk compared to soy milk [26,43]. However, in both these studies, the leucine concentration was suboptimal (<1.8 g) with the protein administered in each study < 20 grams. When the leucine contents of dairy milk and soy milk were matched (2 g), a randomized trial including 48 participants following a 12-week RET program found that consumption of dairy milk or soy milk produced similar increases in lean body mass and strength [9]. Similarly, a recent meta-analysis conducted by Messina et al. concluded that soy protein supplementation produces similar gains in strength and lean body mass in response to RET as dairy milk and whey protein. In terms of dairy and soy milk consumption for post-exercise MPS and resultant changes in muscle mass and strength, the available evidence suggests both kinds of milk will evoke similar anabolic responses provided the leucine threshold is met [8].

Health

There are concerns that soy consumption is associated with an increased risk of breast cancer in women and "feminization" in males [10]; however, the literature does not support these notions. Indeed, in an umbrella review including 114 meta-analyses and systematic reviews examining multiple health outcomes, Li et al. identified beneficial associations for cancers, cardiovascular disease, and gynecological, metabolic, musculoskeletal, endocrine, neurological, and renal outcomes with soy and isoflavone consumption [44]. Considering the positive health associations observed with soy consumption, along with the similarities in protein content and influence on circulating sex hormones between milks, and probable equivalence in facilitating MPS between dairy and soy milk, soy milk consumption should be considered as a viable alternative to dairy milk as a recovery beverage. 

Limitations

While this pilot study provides novel insights into the effects of soy and dairy milk on circulating sex hormones, it is not without limitations. The sample size was small, limiting statistical power to detect differences between milk conditions, and only included men in the age group of 18-30 years, thereby limiting the applicability to women as well as younger and older individuals. Further, the concentrations of sex hormones within the milk provisions were unknown. Assumptions were made relying on published data describing the concentrations of sex hormones within these milks, often obtained from countries outside of Australia, where the milk was sourced for this study. An analysis of the sex hormone constituents within the two types of milk would therefore have strengthened this study. Lastly, without a control group consuming neither soy or dairy milk (e.g. water), it is difficult to isolate the influence of either milk on circulating sex hormones. A third arm consisting of a control group should be considered for future studies.

## Conclusions

This pilot study, the first to investigate the influence of dairy versus soy milk consumption as a post-exercise recovery beverage on circulating sex hormones following RET, revealed no significant differences in circulating serum sex hormones between the two milk consumption conditions. Soy milk consumption did not result in lower circulating testosterone or higher circulating total estrogen than dairy milk consumption. These findings are important since they imply that both dairy milk and soy milk can be considered equally in terms of their effects on sex hormones, with both types of milk providing viable options for promoting MPS and muscle hypertrophy in the context of post-exercise recovery. These results hold relevance for recreational exercisers and elite athletes pursuing increased muscle mass and strength, as they suggest that the choice between dairy milk and soy milk as a recovery beverage is unlikely to meaningfully impact circulating sex hormones. However, given the pilot nature of this study, further research with larger sample sizes is needed to corroborate and expand upon these preliminary findings.

## Appendices

**Table 2:** Descriptive statistics between milk consumption at each time point for total estrogen, free testosterone, total testosterone, free testosterone, progesterone, and SHBG. SHBG: sex hormone binding globulin

		Dairy Milk				Soy Milk								
		Mean	SD	Min	Max	Mean		SD		Min		Max		
Total Estrogen (pg/mL)	Time 0 minutes	87	43	34	161	94		42		42		166		
	Time 30 minutes	88	53	25	178	71		31		30		112		
	Time 45 minutes	65	43	32	137	99		54		37		174		
	Time 60 minutes	74	32	48	126	76		42		30		125		
	Time 80 minutes	65	49	48	140	86		36		48		140		
	Time 100 minutes	75	58	38	186	84		51		15		158		
	Time 120 minutes	62	52	13	161	91		51		32		177		
Free testosterone (ng/mL)	Time 0 minutes	0.15	0.033	0.11	0.19	0.15		0.024		0.12		0.19		
	Time 30 minutes	0.098	0.028	0.064	0.13	0.093		0.022		0.073		0.14		
	Time 45 minutes	0.089	0.027	0.052	0.13	0.09		0.016		0.074		0.12		
	Time 60 minutes	0.092	0.028	0.054	0.13	0.091		0.019		0.073		0.12		
	Time 80 minutes	0.087	0.028	0.055	0.13	0.086		0.012		0.072		0.1		
	Time 100 minutes	0.084	0.03	0.046	0.13	0.082		0.017		0.063		0.11		
	Time 120 minutes	0.089	0.028	0.059	0.13	0.093		0.014		0.078		0.11		
Total testosterone (ng/mL)	Time 0 minutes	7.4	1.9	5.3	10	7.1		1.9		5.6		11		
	Time 30 minutes	4.8	1.8	2.3	7	4.6		1.7		2.7		7.4		
	Time 45 minutes	4.4	1.8	2.2	6.4	4.6		1.3		3.5		6.6		
	Time 60 minutes	4.4	1.6	2.7	6.3	4.6		1.5		3.2		7		
	Time 80 minutes	4.2	1.6	2.2	5.9	4.4		1.2		3.5		6.4		
	Time 100 minutes	4.2	1.7	2.4	6.2	4		1.3		2.9		6.6		
	Time 120 minutes	4.4	1.6	2.6	6.2	4.6		1.2		3.2		6.5		
Free Testosterone (%)	Time 0 minutes	2.1	0.42	1.5	2.7	2.1		0.43		1.6		2.7		
	Time 30 minutes	2.2	0.43	1.6	2.8	2.2		0.56		1.6		3.2		
	Time 45 minutes	2.2	0.51	1.7	3	2.1		0.39		1.6		2.7		
	Time 60 minutes	2.1	0.39	1.7	2.6	2		0.38		1.6		2.6		
	Time 80 minutes	2.2	0.43	1.6	2.7	2		0.39		1.6		2.6		
	Time 100 minutes	2.1	0.39	1.6	2.5	2.1		0.33		1.6		2.6		
	Time 120 minutes	2.1	0.45	1.5	2.6	2.1		0.38		1.6		2.6		
Progesterone (ng/mL)	Time 0 minutes	0.35	0.064	0.24	0.41	0.35		0.1		0.25		0.51		
	Time 30 minutes	0.27	0.041	0.22	0.32	0.34		0.086		0.25		0.5		
	Time 45 minutes	0.29	0.034	0.23	0.33	0.32		0.073		0.24		0.42		
	Time 60 minutes	0.31	0.088	0.23	0.47	0.31		0.063		0.24		0.39		
	Time 80 minutes	0.29	0.073	0.22	0.38	0.22		0.049		0.22		0.34		
	Time 100 minutes	0.26	0.043	0.21	0.32	0.26		0.047		0.21		0.32		
	Time 120 minutes	0.25	0.037	0.2	0.31	0.24		0.045		0.2		0.31		
SHBG (nmol/L)	Time 0 minutes	35	15	19	59	35		16		19		56		
	Time 30 minutes	33	13	19	51	33		14		15		52		
	Time 45 minutes	32	13	16	49	33		13		18		52		
	Time 60 minutes	32	12	19	49	33		13		19		51		
	Time 80 minutes	32	13	17	51	34		14		19		52		
	Time 100 minutes	33	13	18	51	30		11		19		50		
	Time 120 minutes	33	14	18	55	32		13		19		50		
